# Impact of preoperative renal replacement therapy on the clinical outcome of heart transplant patients

**DOI:** 10.1038/s41598-021-92800-0

**Published:** 2021-06-28

**Authors:** Darae Kim, Jin-Oh Choi, Yang Hyun Cho, Kiick Sung, Jaewon Oh, Hyun Jai Cho, Sung-Ho Jung, Hae-Young Lee, Jin Joo Park, Dong-Ju Choi, Seok-Min Kang, Jae-Joong Kim, Eun-Seok Jeon

**Affiliations:** 1 Division of Cardiology, Department of Medicine, Heart Vascular Stroke Institute, Samsung Medical Center, Sungkyunkwan University School of Medicine, 81 Irwon-Ro Gangnam-gu, Seoul, 06351 Republic of Korea; 2 Department of Thoracic and Cardiovascular Surgery, Samsung Medical Center, Sungkyunkwan University School of Medicine, Seoul, Republic of Korea; 3 Department of Internal Medicine, Yonsei University College of Medicine, Seoul, Republic of Korea; 4 Department of Internal Medicine, Seoul National University College of Medicine, Seoul, Republic of Korea; 5 Department of Thoracic Surgery, Asan Medical Center, University of Ulsan College of Medicine, Seoul, Republic of Korea; 6 Division of Cardiology, Department of Internal Medicine, Seoul National University Bundang Hospital, Seongnam, Gyeonggi Republic of Korea; 7 Department of Internal Medicine, Asan Medical Center, University of Ulsan College of Medicine, Seoul, Republic of Korea

**Keywords:** Cardiology, Health care, Risk factors

## Abstract

Renal dysfunction is considered as a relative contraindication for heart transplantation (HTx). However, in the real world setting, many patients with advanced heart failure (HF) experience worsening of renal function and some even require renal replacement therapy (RRT) by the time they undergo HTx. We aimed to investigate the prognosis and clinical outcomes of HTx patients who required RRT during the perioperative period. The Korean Organ Transplant Registry (KOTRY) is a nationwide organ transplant registry in Korea. A total of 501 HTx patients had been prospectively enrolled in the KOTRY registry during 2014–2018. Among the 501 patients, 13 underwent combined heart and kidney transplantation (HKTx). The 488 patients who underwent isolated HTx were grouped according to their pre- and postoperative RRT status. The primary outcome was progression to dialysis-dependent end-stage renal disease (ESRD) after HTx. The secondary outcome was all-cause mortality after HTx. The median follow-up was 22 months (9–39 months). Patients who needed preoperative RRT but were free from postoperative RRT showed comparable overall survival and renal outcome to patients who were free from both pre- and postoperative RRT. In multivariable analysis, preoperative RRT was not associated with progression to ESRD or all-cause mortality after HTx; however, postoperative RRT was a significant predictor for both progression to ESRD and all-cause mortality after HTx. Preoperative creatinine or estimated glomerular filtration rate (eGFR) were not predictive of progression to ESRD after HTx. The present analysis suggests that preoperative RRT requirement does not indicate irreversible renal dysfunction in patients waiting for HTx. However, postoperative RRT was associated with progression to ESRD and mortality after HTx.

## Introduction

Renal dysfunction is often associated with advanced heart failure (HF) in patients listed for heart transplantation (HTx). The 2016 International Society of Heart and Lung Transplantation criteria stated that irreversible renal dysfunction (estimated glomerular filtration rage (eGFR) < 30 mL/min/1.73 m^2^) is a relative contraindication for HTx alone with level C evidence^1^. A previous study using United Network of Organ Sharing (UNOS) data showed that pre-HTx eGFR was independently associated with mortality and end stage renal disease (ESRD) after HTx^2^.

Determining whether the decline of renal function of advanced HF patients is irreversible due to intrinsic renal dysfunction is difficult to determine. GFR is the most commonly used measure of kidney function; however, low GFR does not mean irreversible intrinsic renal dysfunction. No single laboratory test to determine irreversibility of renal function is currently available.

Although renal dysfunction is considered as a relative contraindication for HTx, in the real world, many patients with advanced HF experience worsening of renal function and some even require renal replacement therapy (RRT) by the time they undergo HTx because of co-existing intrinsic renal dysfunction and/or cardiorenal syndrome. Therefore, the aim of this study was to analyze the prognostic significance of preoperative RRT in HTx patients in terms of the prevalence of future dialysis-dependent ESRD and overall mortality after HTx.

## Results

### Baseline characteristics

Between January 2014 and December 2018, a total of 501 HTx patients were registered in this study. Among the 501 patients, 13 underwent combined heart and kidney transplantation (HKTx); all of these patients were on dialysis preoperatively. The remaining 488 patients were grouped according to pre- and post-HTx RRT status as follows: group 1, patients with pre- and post-HTx eGFR > 30 ml/min/1.73 m^2^ who did not require perioperative RRT; group 2, patients who required preoperative RRT but not postoperative RRT; group 3, patients who required postoperative RRT but not preoperative RRT; and group 4, patients who required both pre- and postoperative RRT. All patients were followed up until death and the last follow-up date was June 2019. The median follow-up was 22 months (9–39 months). Among the 488 patients who underwent isolated HTx, 74 were on either continuous renal replacement therapy (CRRT) (n = 62) or conventional hemodialysis (HD) (n = 12) before HTx (groups 2 and 4).

Table 1 shows the baseline characteristics of patients who underwent isolated HTx in relation to perioperative RRT status. Patients from group 1 had the longest waiting list duration, while patients in groups 2 and 4 had relatively short waiting list duration, suggesting that patients in groups 2 and 4 are likely to be more rapidly decompensated HF patients. Group 4 included more patients with ischemic heart disease and fewer patients with idiopathic dilated cardiomyopathy than other groups. Patients who needed preoperative RRT (groups 2 and 4) were more likely to have diabetes mellitus (DM) compared with patients in the other groups, although this difference was not statistically significant. Patients who needed preoperative RRT (groups 2 and 4) were also more likely to need pre-HTx mechanical cardiac support (p < 0.001) and pre-HTx mechanical ventilator (p < 0.001) than patients in the other groups.

**Table 1:** Pre- and postoperative characteristics of patients who underwent isolated HTx according to perioperative RRT support.

Characteristics	Group 1 (n = 361)	Group 2 (n = 17)	Group 3 (n = 53)	Group 4 (n = 57)	P
	PreRRT (−)	PreRRT (+)	PreRRT (−)	PreRRT (+)	
	PostRRT (−)	PostRRT (−)	PostRRT (+)	PostRRT (+)	
Age, years	55 (44–62)	56 (49–65)	53 (40–60)	54 (46–61)	0.283
Male, n (%)	247 (67.9)	40 (74.1)	19 (79.2)	43 (72.9)	0.448
Height, cm	167 (150–171)	167 (163–176)	168 (160–172)	168 (160–172)	0.738
Weight, kg	61.7 (53.8–68.3)	62.9 (55.1–70.0)	61.1 (53.7–70.0)	63.4 (55.0–76.5)	0.674
Body mass index, kg/m^2^	22.2 (20.0–24.5)	22.1 (19.8–24.3)	23.1 (20.3–24.5)	23.5 (19.4–25.8)	0.649
**Etiology of cardiomyopathy**					
Ischemic	65 (18.0)	2 (11.8)	10 (18.9)	19 (33.3)	0.045
Idiopathic dilated	205 (56.8)	10 (58.8)	27 (50.9)	21 (36.8)	0.041
Hypertrophic	24 (6.6)	0 (0)	5 (9.4)	0 (0)	0.104
Valvular heart disease	16 (4.4)	0 (0)	2 (3.8)	(1.8)	0.644
**Comorbidities**					
Hypertension	107 (29.6)	4 (2.7)	16 (30.2)	21 (36.8)	0.660
Diabetes mellitus	95 (26.3)	6 (35.3)	15 (28.3)	22 (38.6)	0.078
Insulin requiring diabetes mellitus	20 (5.5)	2 (11.8)	5 (9.4)	7 (12.3)	0.194
Preoperative echocardiography					
LV EF, %	24 (18–30)	25 (18–32)	21 (18–32)	20 (15–26)	0.922
LV EDD, mm	58 (48–67)	64 (54–71)	67 (50–76)	62 (52–73)	0.586
LA volume index, ml/m^2^	72 (53–95)	61 (50–76)	77 (59–95)	67 (41–83)	0.586
Septal E/e’	18.3 (12.8–24.2)	23.2 (12.6–34.4)	17.8 (14.9–23.0)	20.5 (16.6–29.3)	0.322
RVSP, mmHg	39 (27–51)	46 (33–56)	47 (34.0–63)	44.0 (29.0–55.0)	0.015
PreHTx mechanical cardiac support	77 (21.2)	14 (82.4)	20 (37.2)	44 (77.2)	< 0.001
ECMO	70 (19.4)	13 (76.5)	20 (37.7)	43 (75.4)	< 0.001
LVAD	6 (1.7)	0 (0)	0 (0)	1 (1.8)	0.755
IABP	1 (0.3)	1 (5.9)	0 (0)	0 (0)	0.004
PreHTx CRRT without previous history of RRT	0 (0)	12 (70.6)	0 (0)	46 (80.7)	< 0.001
PreHTx mechanical ventilator	42 (11.6)	12 (70.6)	19 (35.8)	42 (73.7)	< 0.001
Waiting time since HTx enlisting, days	80 (32–170)	18 (7–56)	39 (13–175)	19 (7–55)	0.004

Data were expressed as median (IQR) or number (%).

*RRT* renal replacement therapy, *DM* diabetes mellitus, *LV* left ventricle, *eGFR* estimated glomerular filtration rate, *HTx* heart transplantation, *LV* left ventricle, *EF* ejection fraction, *EDD* end-diastolic dimension, *LA* left atrium, *E* early mitral inflow velocity, *e*′ early mitral tissue Doppler velocity, *RVSP* right ventricular systolic pressure, *ECMO* extracorporeal membrane oxygenation, *LVAD* left ventricular assisting device, *IABP* intra-aortic balloon pump, *RRT* renal replacement therapy, *EF* ejection fraction, *LV* left ventricle, *EDD* end-diastolic dimension, *CRRT* continuous renal replacement.

### Clinical outcome

The clinical characteristics of the four groups post-HTx are described in Supplementary Table 1. Significantly more patients from group 4 experienced primary graft failure compared with patients in the other groups. Right ventricular systolic pressure was higher in patients who were supported with postoperative RRT (group 3 and 4) at 1 month after HTx.

**Figure 1:**
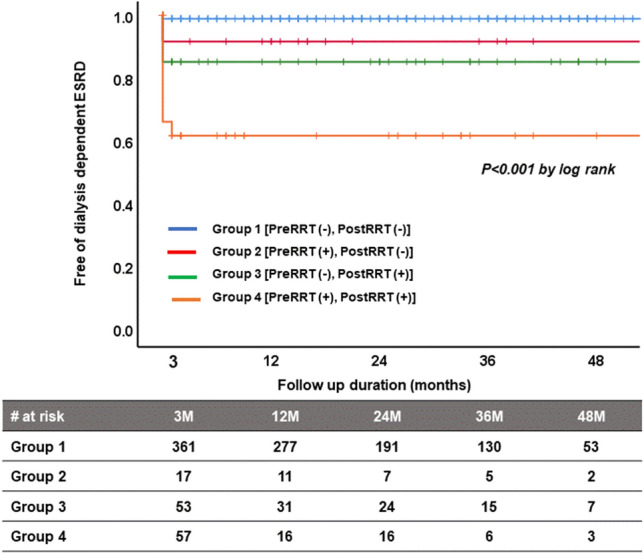
Proportion of patients who developed dialysis-dependent ESRD after HTx in the 4 groups from post-HTx 3 month during follow up. *ESRD* end-stage renal disease, *RRT* renal replacement therapy.

Supplementary Fig. 1 show proportion of patients who required RRT immediately after HTx and at post HTx 3 months. Among the patients who needed early post-HTx RRT (groups 2 and 4), patients from group 2 were less likely to have dialysis-dependent ESRD (11.8%) than those from group 4 (36.8%, p = 0.013). After post HTx 3 month, no patients who became dialysis-dependent ESRD was able to be dialysis free during follow up. Figure 1 shows proportion of patients who became dialysis-dependent ESRD from post-HTx 3 month during follow up. After post-HTx 3 month, significantly more patients in group 4 had dialysis-dependent ESRD (p < 0.001) than patients in the other groups (Fig. 1).

Table 2 shows the renal function of patients who underwent isolated HTx at 6 months and 12 months post-HTx. Patients who were on RRT at 6 month and 12 months were excluded in this table. BUN and creatinine levels were significantly higher in group 4 than in the other groups at both 6 months and 12 months post-HTx. None of patients who developed dialysis-dependent ESRD recovered to be dialysis free.

**Table 2:** Renal function after isolated HTx according to perioperative RRT support.

*N* = *452*	Group 1	Group 2	Group 3	Group 4	P
	PreRRT (−)	PreRRT (+)	PreRRT (−)	PreRRT (+)	
	PostRRT (−)	PostRRT (−)	PostRRT (+)	PostRRT (+)	
BUN at 6-month post-HTx, mg/dL	22.0 (17.0–27.4)	29.0 (24.3–34.8)	24.7 (19.0–30.8)	27.6 (19.9–39.6)	0.007
Creatinine at 6-month post-HTx, mg/dL	1.08 (0.90–1.36)	1.35 (1.13–1.57)	1.27 (0.96–1.49)	1.33 (1.11–1.98)	0.002
eGFR at 6-month post-HTx, ml/min/1.73 m^2^	76.0 (50.9–85.1)	79.2 (61.4–89.5)	67.4 (52.3–87.1)	56.8 (36.0–75.6)	0.021
BUN at 12-month post-HTx, mg/dL	19.9 (15.4–24.0)	28.2 (21.5 39.8)	21.0 (17.1–25.8)	27.0 (21.0–34.5)	0.001
Creatinine at 12-month post-HTx, mg/dL	1.07 (0.87–1.27)	1.50 (1.13–1.69)	1.05(0.91–1.40)	1.36 (1.08–1.81)	0.001
eGFR at 12-month post-HTx, ml/min/1.73 m^2^	79.3 (53.3–88.0)	82.1 (61.0–90.2)	67.9 (45.0–88.9)	54.8 (36.0–75.6)	0.098

Data were expressed as median (IQR).

*BUN* blood urea nitrogen, *eGFR* estimated glomerular filtration rate.

*Patient who were on the dialysis at 6 and 12 months were excluded (n = 452).

Table 3 shows univariate and multivariable analyses for progression to dialysis-dependent ESRD after HTx. In multivariable analysis, insulin-dependent DM and postoperative RRT were significantly associated with progression to ESRD after HTx. However, preoperative RRT was not statistically significant after adjustment of other variables. Supplementary table 2 shows subgroup analysis to predict progression to ESRD after HTx according to preoperative RRT status. The significant clinical predictors for progression to ESRD differed between two subgroups. In the preoperative RRT (−) subgroup, the need for preoperative mechanical ventilation, need for preoperative mechanical cardiac support, baseline eGFR, baseline creatinine, postoperative RRT, and primary graft failure were significant clinical factors in univariate analysis. However, in the preoperative RRT (+) subgroup, insulin-dependent DM and early postoperative RRT were significant clinical predictors.

**Table 3:** Univariable and multivariable analysis to predict progression to dialysis-dependent ESRD after HTx.

Variables	Univariate			Multivariate		
	HR	95% CI	p	HR	95% CI	p
Age, year	1.016	0.988–1.046	0.265	1.009	0.987–1.054	0.385
Male	1.107	0.518–2.367	0.794			
Diabetes mellitus	1.551	0.758–3.173	0.230			
Insulin-dependent diabetes mellitus	4.866	2.013–11.764	< 0.001	2.479	1.085–5.665	0.031
Preoperative mechanical ventilation	4.416	2.188–8.912	< 0.001	0.801	0.296–2.169	0.663
Preoperative mechanical cardiac support	4.072	1.992–8.322	< 0.001	1.612	0.570–4.558	0.368
Preoperative RRT	15.108	7.091–32.186	< 0.001	2.327	0.969–5.648	0.062
Preoperative eGFR, ml/min/1.73 m^2^	0.971	0.957–0.985	0.001	0.994	0.980–1.008	0.410
Preoperative creatinine, mg/dL	2.852	1.844–4.411	< 0.001	1.191	0.883–1.608	0.252
Cardiopulmonary bypass time, minutes	1.006	1.000–1.011	0.040	1.001	0.995–1.007	0.735
Cold ischemia time, minutes	1.000	1.012–1.023	0.956			
Postoperative RRT	3.750	1.694–8.299	< 0.001	8.982	2.928–27.552	< 0.001
Primary graft failure	2.945	1.373–6.316	0.006	0.890	0.374–2.115	0.791

*RRT* renal replacement therapy, *eGFR* estimated glomerular filtration rate, *HTx* heart transplantation, *ESRD* end-stage renal disease.

In addition, we investigated the association between pre-HTx clinical variables and post-HTx renal outcome (Supplementary Table 3). Among pre-HTx clinical variables, from multivariable analysis, insulin-dependent DM and preoperative RRT showed significant associations with progression to ESRD after HTx.

### All-cause mortality after HTx

**Figure 2:**
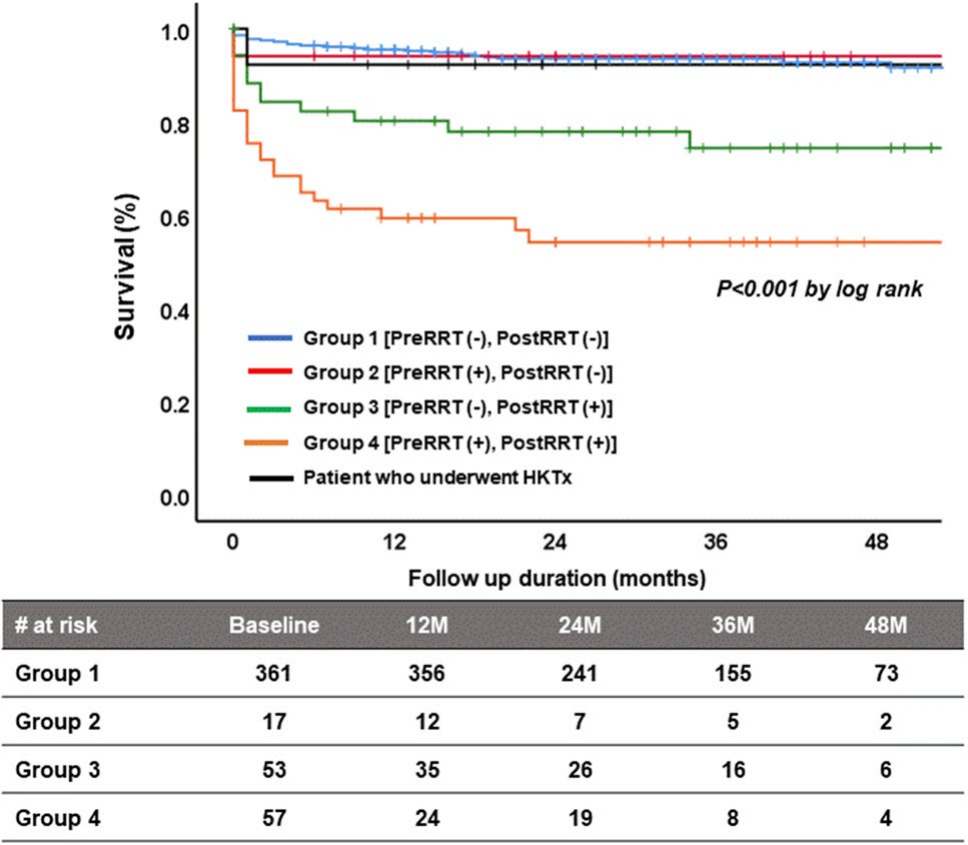
Kaplan–Meier survival curves showing overall survival for patients in the four groups and those who underwent combined HKTx. *RRT* renal replacement therapy, *HKTx* heart and kidney transplantation.

After HTx, patients in group 4 had significantly higher mortality than patients in the other groups (4-year survival rate = 51.9%) (Fig. 2). Patients who underwent combined HKTx showed comparable survival (4-year survival rate = 92.3%) to patients in group 1 (4-year survival rate = 90.3%). Patients in group 2 also showed comparable survival to those in group 1 (4-year survival rate = 94.1%). Table 4 summarizes the univariate and multivariate analyses for all-cause mortality after HTx. From multivariable analysis, postoperative RRT and primary graft failure were the significant predictors of all-cause mortality after HTx. Preoperative eGFR and preoperative creatinine levels were not associated with all-cause mortality after HTx.

**Table 4:** Univariate and multivariate analysis to predict all-cause mortality after HTx.

	Univariate			Multivariate		
	HR	95% CI	p	HR	95% CI	p
Age, year	1.022	0.999–1.039	0.075	1.018	0.988–1.029	0.295
Male	1.193	0.703–2.023	0.514			
DM	1.843	1.136–2.979	0.013	1.749	0.942 – 2.670	0.083
Insulin-dependent DM	1.742	0.723–4.164	0.217			
Preoperative mechanical ventilation	2.874	1.782–4.630	< 0.001	1.394	0.674–2.879	0.893
Preoperative mechanical cardiac support	2.742	1.705–4.410	< 0.001	1.510	0.743–3.048	0.437
Preoperative RRT	2.743	2.302–6.084	< 0.001	1.306	0.723–2.359	0.515
Preoperative eGFR, ml/min/1.73 m^2^	0.993	0.992–1.005	0.588			
Preoperative creatinine, mg/dL	1.044	0.882–1.297	0.753			
Postoperative RRT	6.413	3.949–10.417	< 0.001	4.396	2.469–7.828	< 0.001
ESRD after HTx	5.142	2.464–10.725	< 0.001	1.142	0.708–2.814	0.327
Primary graft failure	2.955	1.373–6.316	0.006	2.434	1.407–4.213	0.001

*DM* diabetes mellitus, *RRT* renal replacement therapy, *eGFR* estimated glomerular filtration rate, *HTx* heart transplantation, *ESRD* end-stage renal disease.

Supplementary Table 4 shows subgroup analysis according to preoperative RRT status. In the preoperative RRT (−) group, from multivariate analysis, early postoperative RRT was the only significant clinical factor. In the preoperative RRT (+) group, early postoperative RRT and primary graft failure were significant predictors for mortality after HTx from multivariate analysis.

## Discussion

The main finding of this study is that preoperative RRT was not associated with progression to ESRD or all-cause mortality after HTx. However, the requirement of post-RRT was a significant predictor for progression to ESRD and all-cause mortality after HTx. Patients who required preoperative RRT but were free from postoperative dialysis showed a comparable prognosis to patients who did not require pre- or postoperative RRT. Perioperative creatinine or eGFR were not predictive of progression to ESRD and all-cause mortality after HTx.

Current ISHLT guidelines have indicated that GFR < 40 ml/min/1.73 m^2^ is a relative contraindication for HTx. This recommendation was level C evidence based on only expert opinion because of the lack of studies linking pre-HTx eGFR with post-HTx outcomes. A study analyzing 30,090 HTx patients using the United Network of Organ Sharing (UNOS) database showed that pre-HTx eGFR was independently associated with mortality and renal outcome after HTx^2^. However, in this study, patients who were on dialysis at the time of HTx were excluded.

Similar to our study, a previous study from a single center produced conflicting results regarding pre-HTx GFR with post-HTx outcomes^3^. Preoperative measured GFR was not predictive of mortality or ESRD after HTx. This is because there is no single laboratory parameter that can determine the irreversibility of renal dysfunction. Our results showed that in patients who needed preoperative RRT, if renal function recovered as a result of improved cardiac function after HTx, preoperative renal function was not predictive of mortality or renal outcome after HTx.

In the real world practice, it is very difficult to specify the etiology of renal dysfunction in advanced HF patients with deteriorating renal function. A notable number of patients experience deterioration of renal function secondary to impaired to cardiac function while waiting for HTx. Our data suggest that postoperative RRT is a significant predictor for all-cause mortality and progression after HTx, while preoperative creatinine, eGFR, or RRT did not show any significant association. However, clinicians need to make decisions about proceeding to HTx based on preoperative data. Distinguishing group 2 patients (preoperative RRT (+)/postoperative RRT (−)) from group 4 patients [preoperative RRT (+)/postoperative RRT (+)] with preoperative clinical characteristics would be useful. However, our results showed that the baseline characteristics of group 2 and 4 patients were similar, except for the higher incidence of idiopathic dilated cardiomyopathy in group 2 compared with group 4.

The 3-month mortality rate on the transplant waiting list for advanced HF patients who are dialysis-dependent is 21%, which is three times higher than the rate for patients not dependent on dialysis^4^. If physicians could determine that the accompanied dialysis-dependent renal dysfunction is irreversible, the best way to improve survival would be combined HKTx^5,6^. However, it is worth noting that the overall survival rate and renal outcome in group 2 were comparable to patients who underwent combined HKTx. This finding suggest that in group 2, compromised renal function that led to RRT was largely due to the hemodynamic consequences of HF, the so-called cardiorenal syndrome, which was reversible with HTx. In clinical practice, it is very difficult to determine the main cause of renal dysfunction and predict the reversibility of renal dysfunction. Therefore, physicians should be very careful when making decisions regarding combined HKTx, because although limited in numbers, some patients show renal recovery with HTx, even though they were on preoperative RRT. Postoperative RRT was a significant predictor of poor survival and renal outcome after HTx. For patients who need postoperative RRT, other interventions, such as delayed kidney transplantation, may be considered to improve outcome.

This study had some limitations. We used eGFR, rather than measured GFR. The exact etiology of renal dysfunction or ESRD, the detailed doses of preoperative inotropic drugs, and other markers of end-organ damage, such as lactate, were not available. In addition, data on the exact duration of the preoperative RTT was not available. However, generally advanced HF patients who are on chronic dialysis (> 3 months) are listed for combined HKTx, and among patients who underwent isolated HTx, most patients who needed preoperative RRT were on CRRT (83%). Therefore, in patients who underwent isolated HTx, most RRT was likely not chronic RRT. Although this is a multi-center study, the follow-up period was relatively short. However, our study results are meaningful, as we described renal outcome after HTx including patients who needed RRT at the time of HTx. These patients were not well represented in previous studies, although many advanced HF patients waiting for HTx already have renal dysfunction. The number of patients is relatively small compared with other national registries. Because a relatively small number of patients showed a follow up of more than 36 months in groups 2 and 4, this suggests a survival bias. In our cohort, high-risk patients who needed preoperative ECMO or mechanical ventilators were included. These patients may not be considered eligible for HTx in other centers.

Despite these limitations, our study provides important findings. Few data describing the clinical outcome of HTx patients who need perioperative RRT are available. Our study is meaningful because our data show the clinical outcome of HTx patients who needed perioperative RRT, and these patients were not often represented in previous data. An important aspect of our study is that this study described a multi-center, nationwide cohort of HTx patients without selection bias that reflects the reality of clinical practice.

## Conclusion

The requirement for preoperative RRT was not associated with all-cause mortality or progression to ESRD after HTx. Patients who were supported with preoperative RRT but free of postoperative dialysis showed comparable survival rates to patients who did not require perioperative RRT. However, postoperative RRT was a significant predictor of progression to ESRD and all-cause mortality after HTx. Preoperative creatinine, eGFR, or RRT status were not associated with post-HTx renal and clinical outcome in the preoperative RRT group. The present analysis showed that preoperative creatinine, eGFR or RRT requirement are not indicative of an irreversible renal dysfunction in patients waiting for HTx. Further studies are needed to determine how to predict the reversibility of renal dysfunction in patients with advanced HF waiting for HTx.

## Methods

### Study design and population

The Korean Organ Transplant Registry (KOTRY) is a web-based, nationwide, organ transplant registry in Korea that was established in 2009^7,8^. The KOTRY consists of active solid organ transplantation centers coordinated by a medical research coordinating center, which validates the data quality and performs regular surveillance of the data collecting process with support from the Korean National Research Institute of Health^9^. For HTx, data were collected from 4 nationally representative hospitals. Each participating center acquired the approval from their Institutional Review Board for the study protocol and prospective acquisition of patient data (Institutional Review Boards of Samsung Medical Center, Yonsei University College of Medicine, Seoul National University College of Medicine, Asan Medical Center, and Seoul National University Bundang Hospital). HTx patients have been prospectively enrolled in the KOTRY registry since 2014. The registry includes baseline demographic data of patients, including age (year) at the transplantation, risk factors, etiology of HF, echocardiographic data, laboratory results including blood urine nitrogen (mg/dL), creatinine (mg/dL) and eGFR (ml/min/1.73 m^2^), and medications, as well as follow-up data, including comorbidities, rejections, hospitalizations, and mortality. Longitudinal follow-up data including post-transplant laboratory results, comorbidities, and mortalities were obtained. Follow-up records were tracked up to patient deaths. To minimize follow-up loss and to maintain the quality of longitudinal data collection, post-transplantation comorbidity and mortality data are collected on regular annual intervals with periodic feedback on each center’s data transfer quality. All methods were carried out in accordance with relevant guidelines and regulations. Informed consent was obtained from all patients and from legally authorized representatives/next of kin for dead patients.

### Renal function estimation

Index creatinine was used to calculate the eGFR using the Chronic Kidney Disease Epidemiology Collaboration (CKD-EPI) equation^10^. Creatinine and blood urea nitrogen (BUN) were measured preoperatively and at 1 month, 6 months, 1 year, and 2 years after HTx. Preoperative BUN, creatinine, and eGFR were assessed one day before HTx.

### Definition and outcomes

Perioperative RRT was defined as the need for dialysis (continuous renal replacement therapy [CRRT] or conventional hemodialysis [HD]). RRT was initiated on the physician’s decision when transplanted patients experienced oliguria (≤ 0.5 mL/kg/h), refractory fluid overload, severe metabolic acidosis (pH < 7.1), or symptoms/signs of uremia. Primary graft dysfunction after HTx was defined as the use of mechanical support within 24 h after the surgery. The primary outcome was progression to dialysis-dependent end-stage renal disease (ESRD) at 3 months post-HTx. The secondary outcome was all-cause mortality after HTx.

### Statistical analysis

Continuous data were expressed as mean ± standard deviation, and categorical variables were expressed as absolute number (percent). Differences in continuous variables between groups were analyzed with Student *t*-tests and Mann–Whitney tests. Differences in categorical values were assessed with Fisher’s exact tests. The proportion of patients who did not develop post-HTx ESRD and post-transplant survival rates were analyzed with the Kaplan–Meier method. Multivariate Cox regression analysis was used to identify independent prognostic factors for all-cause mortality.

All analyses were performed with IBM SPSS Statistics version 25 (SPSS Inc., Chicago, IL, USA). Two-tailed *P* values < 0.05 were considered significant.

## Supplementary Information

Supplementary Information.
