# Supplementary material for: Impact of preoperative renal replacement therapy on the clinical outcome of heart transplant patients
Source: Sci Rep. 2021 Jun 28;11:13398. doi: 10.1038/s41598-021-92800-0 (PMC8239032; doi:10.1038/s41598-021-92800-0)

**Impact of preoperative renal replacement therapy on the clinical outcome of heart transplant patients**

Darae Kim, MD, PhD^1^; Jin-Oh Choi, MD, PhD^1^*, Yang Hyun Cho, MD, PhD^2^, Kiick Sung, MD, PhD^2^, Jaewon Oh, MD^3^,Hyun Jai Cho, MD, PhD^4^; Sung-Ho Jung, MD^5^; Hae-Young Lee, MD, PhD^4^, Jin Joo Park, MD, PhD^6^; Dong-Ju Choi, MD, PhD^6^; Seok-Min Kang, MD, PhD^3^, Jae-Joong Kim, MD, PhD^6^, Eun-Seok Jeon, MD,PhD^1^

**Supplementary table 1. Postoperative clinical characteristics**

LV, left ventricle; EF, ejection fraction; EDD, end-diastolic dimension; LA , left atrium; E, early mitral inflow velocity; e’, early mitral tissue Doppler velocity

Data were expressed as median (IQR) or number (%).

| n=488 | Group 1 | Group 2 | Group 3 | Group 4 | P |
| --- | --- | --- | --- | --- | --- |
|  | PreRRT (-)  PostRRT (-) | PreRRT (+)  PostRRT (-) | PreRRT (-)  PostRRT (+) | PreRRT(+)  PostRRT(+) |  |
| Post-HTx 1month echocardiography |  |  |  |  |  |
| LV EF, % | 66 (58-70) | 66 (58-70) | 65 (52-68) | 64 (61-68) | 0.207 |
| LV EDD, mm | 43.8 (42.9-48.3) | 43.8 (42.9-48.3) | 47.3 (42.6-50.6) | 47.9 (43.0-51.0) | 0.488 |
| LA volume index, ml/m^2^ | 45 (39-54) | 45 (36-55) | 44 (38-49) | 39 (35-46) | 0.877 |
| Septal E/e’ | 13 8(10.0-21.1) | 16.1(11.3-22.5) | 11.6 (10.0-15.3) | 10.7 (8.4-14.7) | 0.347 |
| RVSP, mmHg | 27 (23-34) | 25 (21-32) | 35 (29-43) | 30 (24-40) | <0.001 |
| Immunosuppressive drug at post-HTx 6month |  |  |  |  |  |
| Everolimus | 115 (31.8) | 7(41.2) | 16 (30.2) | 19 (33.3) | 0.805 |
| Tacrolimus | 338 (93.6) | 16 (94.1) | 49 (92.5) | 54 (94.7) | 0.654 |

**Supplementary Table 2. Uni and multivariate analysis to predict progression to ESRD after HTx according to preoperative RRT status.**

| **Preop RRT (-) subgroup (n= 471)** | Univariate | | | | Multivariate | | |
| --- | --- | --- | --- | --- | --- | --- | --- |
| Variables | HR | | 95% CI | p | HR | 95% CI | p |
| Age, year | | 0.991 | 0.950-1.033 | 0.660 | 0.998 | 0.945-1.041 | 0.850 |
| Male | | 1.370 | 0.365-5.145 | 0.641 |  |  |  |
| Diabetes mellitus | | 1.396 | 0.412-4.732 | 0.592 |  |  |  |
| Insulin dependent diabetes mellitus | | 1.075 | 0.395-2.928 | 1.075 |  |  |  |
| Preoperative mechanical ventilation | | 6.309 | 1.964-20.263 | 0.002 | 2.006 | 0.250-16.077 | 0.512 |
| Preoperative mechanical cardiac support | | 4.853 | 1.504-15.695 | 0.008 | 2.301 | 0.307-17.265 | 2.301 |
| Preoperative eGFR, ml/min/1.73 m^2^ | | 0.979 | 0.957-1.001 | 0.006 | 1.002 | 0.983-1.022 | 0.816 |
| Preoperative creatinine, mg/dL | | 2.077 | 1.198-3.600 | 0.009 | 2.306 | 1.209-4.397 | 0.011 |
| Cardiopulmonary bypass time, minutes | | 1.003 | 0.993-1.012 | 0.591 |  |  |  |
| Cold ischemia time, minutes | | 0.991 | 0.979-1.003 | 0.150 |  |  |  |
| Postoperative RRT | | 11.11 | 3.38-36.464 | <0.001 | 9.481 | 2.370-37.922 | 0.001 |
| Primary graft failure | | 4.286 | 1.238-14.840 | 0.022 | 1.699 | 0.392-7.104 | 0.488 |
| **Preop RRT (+) subgroup (n= 87)** | | Univariate | | | Multivariate | | |
| Variables | | HR | 95% CI | p | HR | 95% CI | p |
| Age, year | | 1.024 | 0.982-1.069 | 0.264 | 1.021 | 0.963-1.021 | 0.358 |
| Male | | 0.827 | 0.290-2.356 | 0.722 |  |  |  |
| Diabetes mellitus | | 1.016 | 0.392-2.639 | 0.973 |  |  |  |
| Insulin dependent diabetes mellitus | | 5.011 | 2.517-11.641 | <0.001 | 3.758 | 0.943-14.972 | 0.061 |
| Preoperative mechanical ventilation | | 0.550 | 0.210-1.439 | 0.223 |  |  |  |
| Preoperative mechanical cardiac support | | 0.800 | 0.292-2.195 | 0.665 |  |  |  |
| Preoperative eGFR, ml/min/1.73 m^2^ | | 0.992 | 0.979-1.006 | 0.250 |  |  |  |
| Preoperative creatinine, mg/dL | | 0.994 | 0.744-1.329 | 0.970 |  |  |  |
| Cardiopulmonary bypass time, minutes | | 0.407 | 0.995-1.012 | 0.407 |  |  |  |
| Cold ischemia time, minutes | | 1.001 | 0.998-1.001 | 0.504 |  |  |  |
| Postoperative RRT | | 7.237 | 1.562-33.521 | 0.011 | 8.736 | 1.765-43.227 | 0.008 |
| Primary graft failure | | 1.075 | 0.395-2.925 | 0.887 |  |  |  |

eGFR, estimated filtration rate; RRT, renal replacement therapy

**Supplementary Table 3. PreHTx clinical variable to predict progression to ESRD after HTx.**

|  | Univariate | | | Multivariate | | |
| --- | --- | --- | --- | --- | --- | --- |
| Variables | HR | 95% CI | p | HR | 95% CI | p |
| Age, year | 1.016 | 0.988-1.046 | 0.265 | 0.959 | 0.903-1.018 | 0.172 |
| Male | 1.107 | 0.518-2.367 | 0.794 |  |  |  |
| Diabetes mellitus | 1.551 | 0.758-3.173 | 0.230 |  |  |  |
| Insulin dependent diabetes mellitus | 4.866 | 2.013-11.764 | <0.001 | 5.455 | 1.243-23.552 | 0.023 |
| Preoperative mechanical ventilation | 4.416 | 2.188-8.912 | <0.001 | 0.287 | 0.065-1.258 | 0.098 |
| Preoperative mechanical cardiac support | 4.072 | 1.992-8.322 | <0.001 | 0.180 | 0.065-1.258 | 0.287 |
| Preoperative RRT | 15.108 | 7.091-32.186 | <0.001 | 16.695 | 2.730-45.102 | 0.002 |
| Preoperative eGFR, ml/min/1.73 m^2^ | 0.971 | 0.957-0.985 | 0.001 | 0.970 | 0.937-1.001 | 0.081 |
| Preoperative creatinine, minutes | 2.852 | 1.844-4.411 | <0.001 | 0.926 | 0.623-1.377 | 0.705 |

**Supplementary Table 4. Uni and multivariate analysis to predict all-cause mortality after HTx.**

| **Preop RRT (-) subgroup (n= 471)** | Univariate | | | | Multivariate | | | |
| --- | --- | --- | --- | --- | --- | --- | --- | --- |
| variables | | HR | 95% CI | p | | HR | 95% CI | p |
| Age, year | | 1.028 | 0.999-1.057 | 0.059 | | 1.013 | 0.989-1.024 | 0.059 |
| Male | | 1.220 | 0.588-2.532 | 0.593 | |  |  |  |
| Diabetes mellitus | | 1.481 | 0.740-2.964 | 0.268 | |  |  |  |
| Insulin dependent diabetes mellitus | | 1.474 | 0.452-4.806 | 0.520 | |  |  |  |
| Preoperative mechanical ventilation | | 1.780 | 0.810-3.913 | 0.151 | |  |  |  |
| Preoperative mechanical cardiac support | | 1.888 | 0.941-3.788 | 0.074 | |  |  |  |
| Preoperative eGFR, ml/min/1.73 m^2^ | | 0.995 | 0.985-1.006 | 0.365 | |  |  |  |
| Preoperative creatinine, mg/dL | | 0.736 | 0.482-1.125 | 0.157 | |  |  |  |
| Early postoperative RRT | | 3.933 | 1.966-7.869 | <0.001 | | 2.978 | 1.375-6.450 | 0.006 |
| ESRD after HTx | | 4.723 | 1.666-13.389 | 0.003 | | 2.174 | 0.697-6.779 | 0.181 |
| Primary graft failure | | 2.745 | 1.250-6.027 | 0.012 | | 1.828 | 0.792-4.220 | 0.157 |
| **Preop RRT (+) subgroup (n= 87)** | | Univariate | | | | Multivariate | | |
| variables | | HR | 95% CI | p | | HR | 95% CI | p |
| Age, year | | 1.007 | 0.975-1.041 | 0.660 | | 1.021 | 0.985-1.038 | 0.660 |
| Male | | 1.027 | 0.434-2.429 | 0.952 | |  |  |  |
| Diabetes mellitus | | 1.425 | 0.669-3.036 | 0.358 | |  |  |  |
| Insulin dependent diabetes mellitus | | 1.356 | 0.513-3.583 | 0.539 | |  |  |  |
| Preoperative mechanical ventilation | | 2.085 | 0.841-5.168 | 0.113 | |  |  |  |
| Preoperative mechanical cardiac support | | 1.606 | 0.648-3.980 | 0.307 | |  |  |  |
| Preoperative eGFR , ml/min/1.73 m^2^ | | 0.996 | 0.989-1.004 | 0.374 | |  |  |  |
| Preoperative creatinine, mg/dL | | 1.041 | 0.889-2.079 | 0.157 | |  |  |  |
| Early postoperative RRT | | 13.887 | 1.883-62.421 | 0.010 | | 10.340 | 1.380-77.482 | 0.023 |
| ESRD after HTx | | 1.794 | 0.832-3.867 | 0.775 | | 1.800 | 0582-5.556 | 0.307 |
| Primary graft failure | | 3.996 | 1.846-8.469 | <0.001 | | 2.901 | 1.331-6.234 | 0.007 |

eGFR, estimated filtration rate; RRT, renal replacement therapy; ESRD, end-stage renal disease; HTx, heart transplant

**Supplementary Figure 1**. A summary of renal outcome from immediately after post-HTx to post-HTx 3 months.


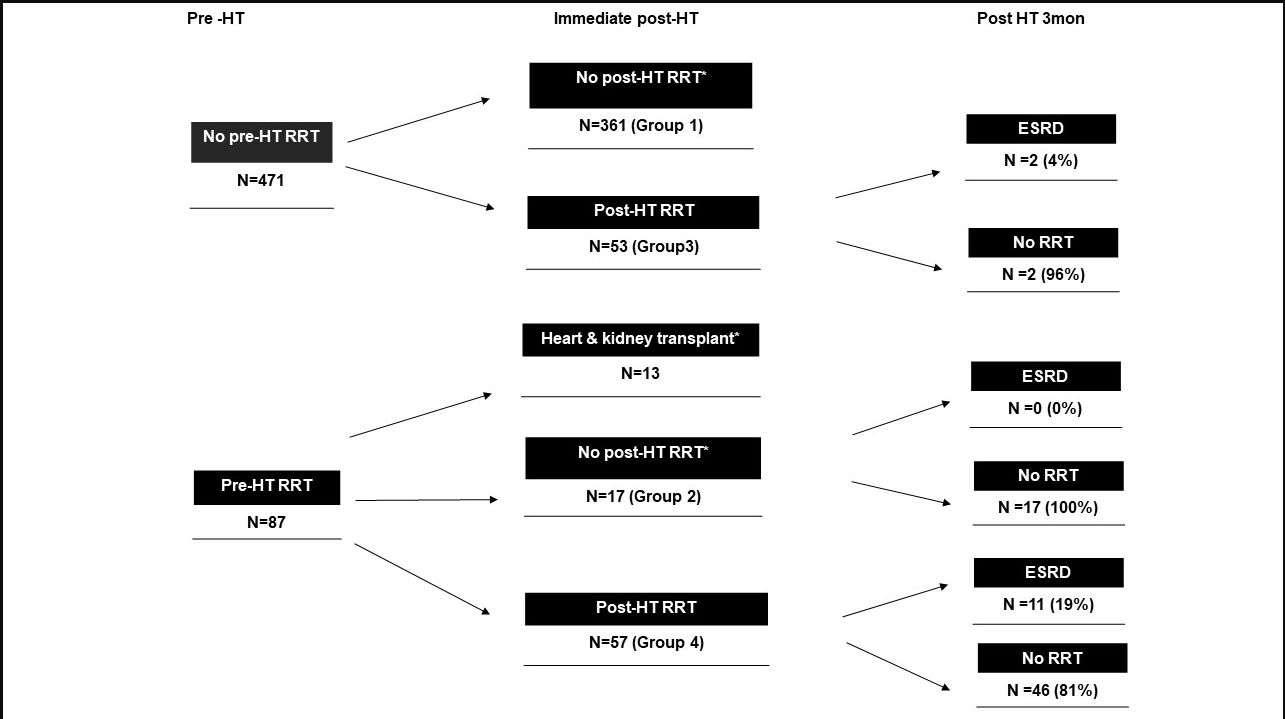

Supplement: Supplementary file 1 — Supplementary Information. [file 41598_2021_92800_MOESM1_ESM.docx]
